# Three‐dimensional quantification of the relationship between the upper first molar and maxillary sinus

**DOI:** 10.1002/cre2.561

**Published:** 2022-03-25

**Authors:** Tobias Regnstrand, Mostafa Ezeldeen, Sohaib Shujaat, Khalid Ayidh Alqahtani, Daniel Benchimol, Reinhilde Jacobs

**Affiliations:** ^1^ Section of Oral Diagnostics and Surgery, Department of Dental Medicine, Division of Oral Diagnostics and Rehabilitation Karolinska Institutet Huddinge Sweden; ^2^ OMFS‐IMPATH Research Group, Department of Imaging and Pathology, Faculty of Medicine Catholic University of Leuven Leuven Belgium; ^3^ Department of Oral and Maxillofacial Radiology Prince Sattam Bin Abdulaziz University Al Kharj Saudi Arabia

**Keywords:** anatomy, cone beam computed tomography, maxillary sinus, molar

## Abstract

**Objectives:**

The present study aims to describe the relationship between upper first molar roots and maxillary sinus, for the first time with a truly three‐dimensional approach.

**Methods:**

From a retrospective cone‐beam computed tomography (CBCT) sample of the upper jaw, a total of 105 upper first molars in contact with maxillary sinus from 74 patients (male 24, female 50, mean age 42) were included in the present study. Segmentation of the upper first molar and maxillary sinus in CBCT was performed utilizing a semiautomatic livewire segmentation tool in MeVisLab v.3.1. The segmentations were analyzed in 3‐matic Medical 20.0 for root volume and the contact area between upper first molar roots and maxillary sinus. Analysis of variance test was applied to detect statistically significant differences between the roots.

**Results:**

The palatal root had the largest contact area with maxillary sinus 27.8 ± 21.4 mm^2^ (20% of the root area) followed by the mesiobuccal 20.5 ± 17.9 mm^2^ (17% of the root area) and distobuccal root 13.7 ± 12 mm^2^ (14% of the root area). A significant difference in the contact area of the different roots of the upper first molar was seen.

**Conclusions:**

This study showed that 70% of the upper first molars were in contact with the maxillary sinus. The palatal root had on average a fifth of its root surface in contact with the sinus, while for mesiobuccal this was a sixth of its root surface and distobuccal roots this was somewhat less. The true 3D relationship could help to better understand maxillary anatomy in relation to occurring pathologies and treatment planning in this area.

## 1 INTRODUCTION

A thorough knowledge of the anatomical relationship between upper posterior teeth and maxillary sinus (MS) is vital for clinicians to allow proper diagnosis in the posterior maxilla as well as to prevent complications while performing dental procedures, such as apical surgery, tooth extraction, endodontic treatment, and implant placement. Such prior knowledge should be obtained via a meticulous radiological examination of the relationship between upper teeth and MS. This is particularly important for diagnosis as well as for treatment planning of upper molars. This holds surely true for the upper first molar, considering that it is one of the teeth with the closest relationship to MS, while being more often associated with odontogenic sinusitis and oro‐antral communications (Maillet et al., 2011; Punwutikorn et al., 1994).

Most of the previous studies assessing the relationship between upper teeth and MS have utilized two‐dimensional (2D) imaging. However, 2D imaging offers limited information as it compresses the 3D structures, leading to misinterpretation of the relation between upper teeth and MS with a potential risk for misdiagnosis of pathology associated with the posterior maxilla (Estrela et al., 2016; Kilic et al., 2010; Nino‐Barrera et al., 2018; Regnstrand et al., 2021; Shahbazian et al., 2014, 2015; von Arx et al., 2014). Three‐dimensional imaging using cone‐beam computed tomography (CBCT) may offer a solution to better visualize the complex representation of MS in relation to upper (pre)molars. Furthermore, CBCT allows a precise diagnostic tool in assessing sinusitis with a possible dental cause (Lofthag‐Hansen et al., 2007). Additionally, with the advent of the state‐of‐the‐art software programs for image analysis, a precise anatomical relationship based on CBCT volumetric data can be further quantified. Even though CBCT imaging may offer a higher diagnostic potential, studies focusing on teeth and MS relationships have so far mostly focused on either linear or angular measurement techniques in different sectional planes. These methodologies are pseudo‐3D and do not provide additional information related to the true 3D relationship. Furthermore, no studies have been carried out quantifying the true 3D relationship between upper teeth and MS.

Therefore, the present study was conducted to determine the true 3D relationship of the upper molar and MS that could help to better understand the 3D anatomy in the posterior maxilla. In this particular study, we opted to unravel the relation of MS and first upper molar considering that the frequency of MS relation, the occurrence of associated pathology, and related surgical complexity are higher for this tooth (Gürhan et al., 2020).

## 2 MATERIALS AND METHODS

### 2.1 Data acquisition

A retrospective sample of 380 CBCT scans of patients referred to the University Hospital of Leuven was reviewed, following ethical approval by the Clinical Trial Center and the Ethical Committee of the Catholic University of Leuven and the University Hospital of Leuven (reference number: S57587). Of these, 147 CBCT scans covering the area of the upper first molar were assessed in this study. The samples were recruited from the Dentomaxillofacial Radiology Center at the University Hospital of Leuven from the first 6 months of 2013. Because of the retrospective approach of this project, no informed consent from the patients was obtained. Images were acquired upon referral for implant planning, orthognathic surgery, endodontic treatment, and assessment of maxillofacial pathogenesis. All scans were acquired using the 3D Accuitomo 170® device (3D Accuitomo; J. Morita, Kyoto, Japan) operating at 90 kV and 5 mA with an exposure time of 17.5 s and field of view (FOV) ranging between 6 × 6 cm and 17 × 17 cm. Voxel size varied between 0.125 and 0.250 mm^3^. Inclusion criteria involved scans exhibiting upper first molars and MS floor and teeth with normal root configuration (mesiobuccal, distobuccal, and palatal root). From a total of 147 CBCT scans, 74 CBCT scans contained upper first molars with normal root configuration in contact with MS and were therefore included in the study. Contact was defined as the fusion of lamina dura of roots with the cortical lining of MS. Patients having a history of a maxillary dental implant, sinus augmentation, oro‐antral communication, maxillofacial trauma, orthognathic surgery, and low‐quality images with metal and/or motion artifacts were excluded. In addition, upper first molars presenting severe periodontitis (with more than half of the length of the root), uncommon root configuration, and periapical lesion were also excluded.

### 2.2 Segmentation protocol

**Figure 1:**
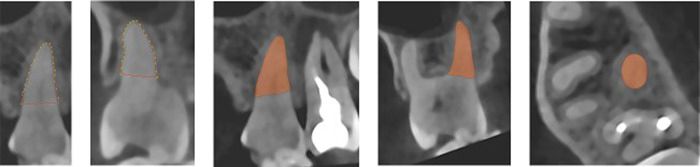
Segmentation and control of the accuracy of the suggested root shape in the three orthogonal planes (sagittal, coronal, and axial) in comparison to the root shape in the CBCT image. CBCT, cone‐beam computed tomography

**Figure 2:**
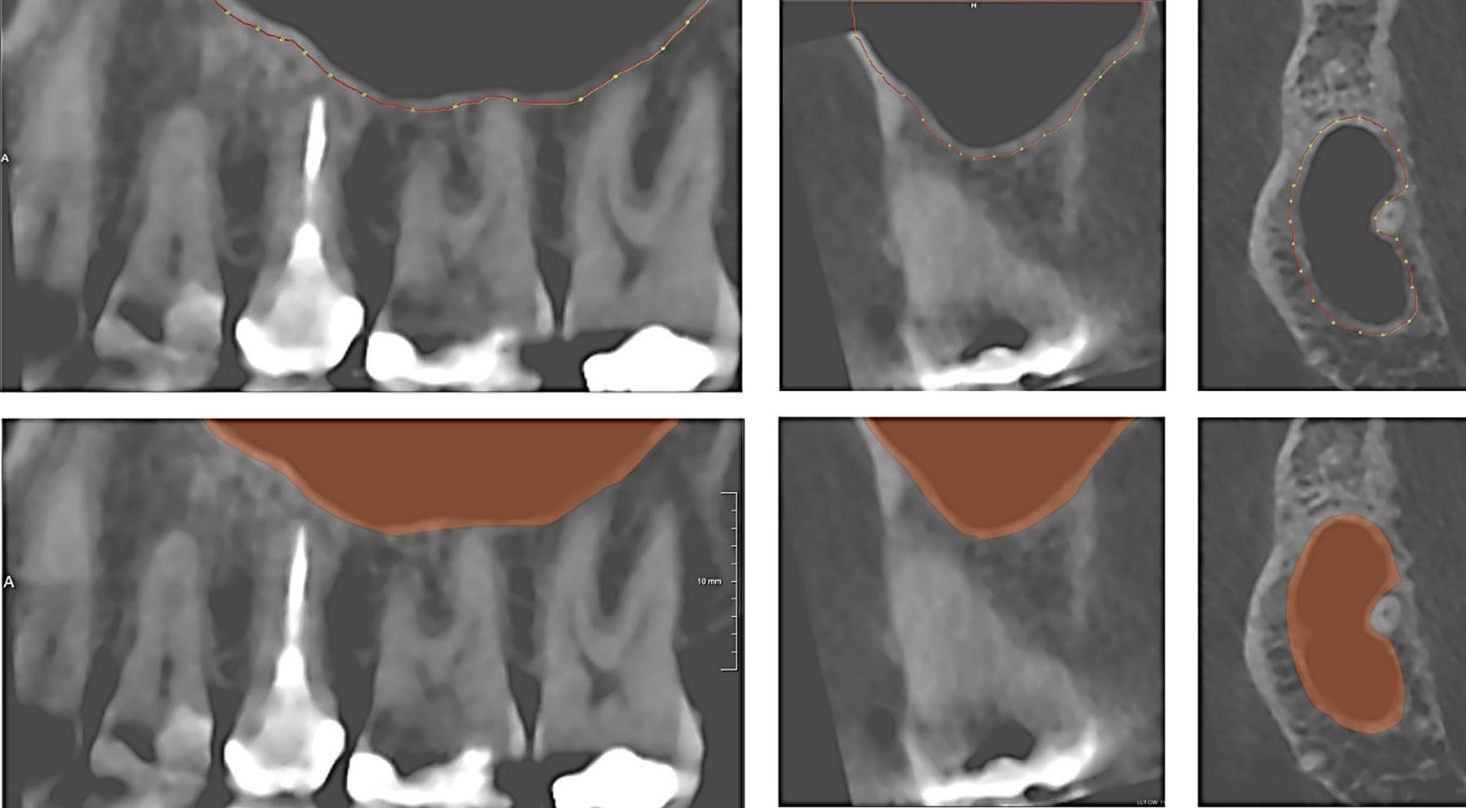
Segmentation of maxillary sinus and control of the suggested shape of the maxillary sinus in the three orthogonal planes (sagittal, coronal, and axial) in comparison to the shape of the maxillary sinus in the CBCT image. CBCT, cone‐beam computed tomography

Roots of the upper first molar in relation to the MS floor were segmented using a validated segmentation framework developed in MeVisLab (MeVis Medical Solutions AG, Bremen, Germany) (Ezeldeen et al., 2015). A semiautomatic user‐guided livewire boundary extraction tool was used to mark the borders of the periodontal ligament space of the root and outer limit of the cortical border of the MS floor adjacent to the first molar (Figures 1 and 2). The livewire segmentation tool enabled precise extraction of the borders (Barrett & Mortensen, 1997; Yushkevich et al., 2006). Segmentation was then compared subjectively with the actual shape of the structures in axial, coronal, and sagittal views of the CBCT scan to ensure accuracy of the segmentation in different orthogonal planes (Figure 1). Each root was segmented with a cut perpendicular to the longitudinal axis of the root at the bifurcation area. Thereafter, segmented structures were saved and exported as Standard Triangle Language (STL) files.

### 2.3 Contact area and volume quantification

**Figure 3:**
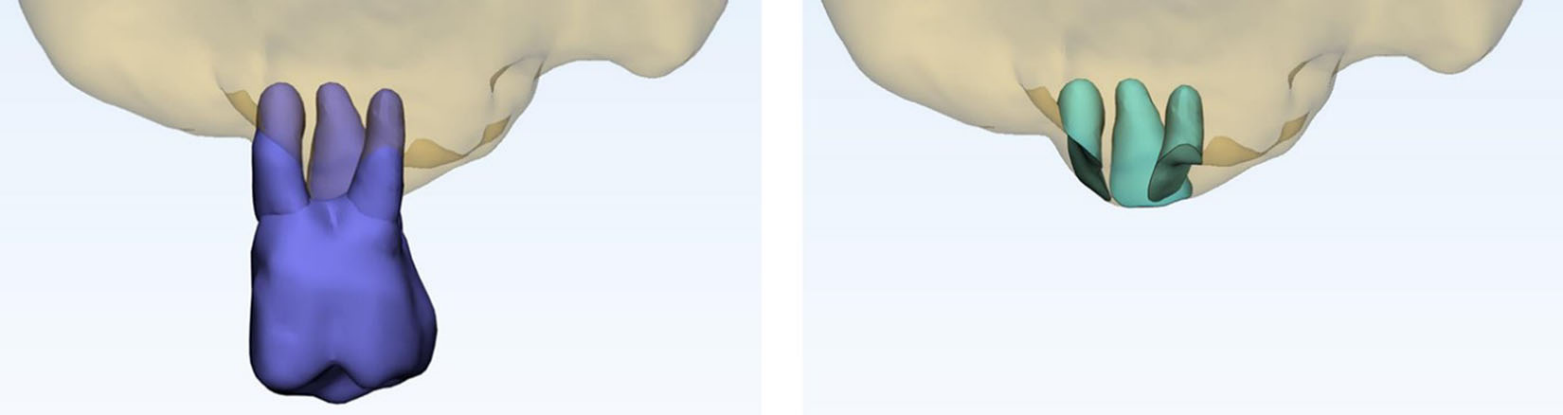
Fusion of the root and sinus segmentations (left). The contact area and contact volume are assessed from the parts of the roots in contact with the maxillary sinus (right)

The segmented roots and MS floor were imported to 3‐matic Medical 20.0 (Materialize N.V., Leuven, Belgium) for quantifying the 3D relationship between the roots and sinus. Following visualization, the contact area between the segmentation of the root and MS was calculated automatically by the software program. Data of the overlapping volume between segmented roots and MS, also described as root volume in contact with MS, were also collected (Figure 3).

The methodology was repeated for 10% of the cases twice by two observers blindly at an interval of four months following initial training and calibration.

### 2.4 Statistical analysis

Statistical analyses were performed with S‐plus 8.0 for Linux (Tibco software, Palo Alto, CA, USA). The mean and percentage of contact area and volume were calculated for assessing the individual and combined relationship of mesiobuccal (MB), distobuccal (DB), and palatal root (P) with the MS. A one‐way analysis of variance (ANOVA) test was applied to detect a statistically significant difference (*p* < .05) for both contact area and volume between the roots and MS. *p* Values were adjusted for multiple comparisons according to Tukey. The relation between age and contact area was assessed by a weighted linear mixed model with age as an explanatory variable and contact area as a continuous variable. Tooth, nested in patient, was used as a random variable. A weighing was applied that was proportional to the age. Inter‐ and intra‐observer agreement was calculated using the intra‐class correlation coefficient (ICC).

## 3 RESULTS

In total, 147 CBCTs covering the area of the upper first molar were assessed, one per patient (87 female and 59 male patients). After applying the inclusion criteria, 74 patients and their CBCT scans were included (50 women, 24 men; mean age 42 years, range 17–81 years). CBCT scans had different FOV, 31 out of 74 was covering both sides of the upper jaw with two first molars in the same patient, while 43 CBCT scans covered one side and one upper molar. On the 147 CBCT scans  213 upper first molars were depicted, of which of these 108 had to be excluded (*n* = 32 no contact between tooth and MS, *n* = 34 apical periodontitis, *n* = 20 uncommon root configuration, *n* = 22 low image quality). In total, 105 first molars (16: *n* = 51, 26: *n* = 54) in contact with MS from 50 female and 24 male patients were included. From these included first molars, a total of 287 roots were in contact with the MS (MB: *n* = 96, DB: *n* = 91 and P: *n* = 100). Contact area ICC for inter‐ and intra‐observer reliability was 0.91 and 0.82, respectively. Contact volume ICC for inter‐ and intra‐observer reliability was 0.83 and 0.85, respectively.

**Figure 4:**
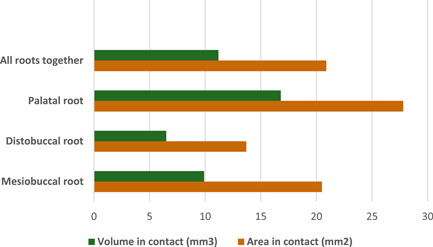
Mean contact area and mean contact volume for different root types

**Figure 5:**
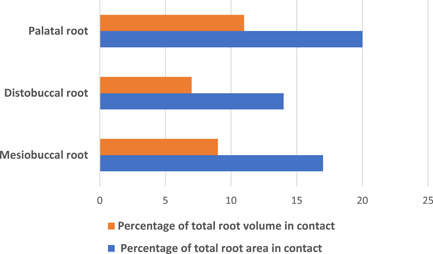
Percentage of root area and root volume in contact with maxillary sinus

The P root showed the largest mean contact area (27.8 ± 21.4 mm^2^) followed by the MB (20.5 ± 17.9 mm^2^) and DB root (13.7 ± 12 mm^2^) (Figure 4). In relation to percentage, P root showed 20% of the root area in contact with the MS, followed by MB (17%) and DB (15%) (Figure 5). Both mean and percentage values showed a significant difference related to the contact area between the DB‐MB, DB‐P, and MB‐P roots (*p* < 0.05) (Table 1).

**Table 1:** Comparison of differences in the area (mm^2^) and volume (mm^3^) between the roots of the upper first molar and their intimate relationship to the maxillary sinus

Root comparison	Contact area	Contact volume	% root area in contact	% root volume in contact
Distobuccal root—Mesiobuccal root	13.7–20.5	6.5–9.9	13.7–15.7	7.5–8.7
	*p* ≤ .001	*p* ≤ .001	*p* = .04	*p* = .037
Distobuccal root—Palatal root	13.7–27.8	6.5–16.7	13.7–19.4	7.5–11.2
	*p* ≤ .001	*p* ≤ .001	*p* ≤ .001	*p* ≤ .001
Mesiobuccal root— Palatal root	20.5–27.8	9.9–16.7	15.7–19.4	8.7–11.2
	*p* = .003	*p* = .005	*p* = .005	*p* = .037

The P root had the largest mean volume in contact with MS (16.7 ± 18.1 mm^3^) followed by the MB (9.9 ± 9.9 mm^3^) and DB root (6.5 ± 8.1 mm^3^) (Figure 4) (Table 1). The percentages of root volume in contact with MS were highest for P root (11%) (Figure 5). The contact volume differed significantly for all roots (Table 1). Gender differences were not significant, with men showing a mean contact area of 24 mm^2^ and women 19 mm^2^ (*p* = .08), with an average contact volume of 14 mm^3^ and 10 mm^3^, respectively (*p* > .05). Furthermore, age did not have an influence on contact volume (*p* = .57) nor contact area (*p* = 0.51).

## 4 DISCUSSION

Dentomaxillofacial anatomy in the maxillary region is complex, thereby creating challenges for diagnosis and therapy. While dentists typically use intraoral radiographs, one should realize the limitations of a 2D approach for visualizing and interpreting complex anatomical structures. In intraoral images, the MS often projects over the roots, which leads to underdiagnosis of approximately 60% of the apical lesions. Of the missed apical lesions, over 40% are found in the first molar region (Shahbazian et al., 2015). Previous studies using CBCT assessed the relationship between MS and proportional or linear root protrusion into the sinus. Yet, to fully benefit from the information available in CBCT, one should maximize the information present in the image and allow for a true 3D approach to assess this complex or intimate 3D relationship. This allows a more thorough understanding of how structures relate to each other, improving risk assessment and treatment planning.

The resolution of the CBCT scans in the current study varied between 0.15 and 0.250 mm^3^. Previous studies suggest no significant difference related to 3D analysis up to a voxel size of 0.3 mm^3^ (Maret et al., 2012, 2014; Sang et al., 2016). At the same instance, Maret et al. (2012) found 0.2 mm^3^ voxel size to be more accurate compared to 0.3 mm^3^. However, we found a good agreement between the observers irrespective of the voxel size.

For the assessed roots included in the present study (roots in contact with MS), 17% of the total root surface area was in contact with the MS. Roots positioned in the cortical border of MS take a longer time to orthodontically move and have an increased risk of root resorption compared to roots positioned in the cancellous bone (Wehrbein et al., 1990). It might be hypothesized that such an intimate relationship might be partially responsible for delayed tooth movement and root resorption and even be involved in primary eruption failure in the posterior upper jaw. Pneumatization of sinus and sinus floor remodeling may have an influence on the intimate relation between the sinus and roots of the upper posterior teeth. Although Torres et al. (2017) reported that the size of MS increased wixth age, the present study did not find any age influences on the relationship between the first upper molar and MS. The study did not find that gender had an influence on the relationship of the upper first molar and MS, which corresponds to previous studies (Kilic et al., 2010; Ok et al., 2014). A limitation in the present study is the rather small number of patients in the subgroups when comparing genders. Future studies assessing differences between the genders should consider assessing a larger sample size. Luz et al. (2018) reported however that men had a generally larger sinus volume thanwomen. From all first molars (213) depicted in CBCT (n = 213), the number of first molars in contact with MS was found to be very high, exceeding up to 72%. This findings were in accordance with previous studies. (Jung & Cho, 2012; Shahbazian et al., 2014; Tian et al., 2016).

From all first molars (213) depicted in CBCT (n = 213), the number of first molars in contact with MS was found to be very high, exceeding up to 72%. Our findings were in accordance with previous studies showing a high percentage of first molars in contact with MS (Jung & Cho, 2012; Shahbazian et al., 2014; Tian et al., 2016).

The palatal root of the first upper molar is most often involved in oro‐antral communication (Punwutikorn et al., 1994). Our findings indicated the presence of a large contact area of the palatal root in relation to the MS, which might help to understand and provide additional information related to the radiological behavior of oro‐antral communication in further studies. Future studies in this area should benefit from the use of 3D analysis for a more thorough investigation.

Our study was limited to the assessment of first molars with normal root configuration. Future studies should be carried out utilizing the present methodology to assess the relationship of all posterior teeth with the MS. Since the complete sinus was not segmented, considering the retrospective nature of the study and the related ALARA principle, analyzing the effect of  root–MS relationship on the entire volume and dimensions of the MS was not possible to obtain.

## 5 CONCLUSION

In conclusion, the present study provided evidence related to the true 3D relationship of the upper first molar roots and the MS. This information could help to better understand the 3D maxillary and dentoalveolar anatomy, the occurring pathologies, and related treatment complexity in the posterior maxilla. Most of the upper first molars were found to be in contact with MS with the palatal root having the largest contact and surface area followed by the mesiobuccal and distobuccal roots. These findings are important to take into consideration for clinical and (2D) radiological diagnosis of sinusitis with a dental origin as well as for dental treatment or oral and maxillofacial surgical procedures in the MS region.

## AUTHOR CONTRIBUTIONS

*Study design, data acquisition, data analysis, data interpretation, manuscript drafting, approval of the final version*: Tobias Regnstrand. *Study design, data analysis, manuscript revising, approval of the final version*: Mostafa Ezeldeen. *Study design, data acquisition, manuscript revising, approval of the final version*: Sohaib Shujaat and Khalid A. Alqahtani. *Study design, data interpretation, manuscript revising, approval of the final version*: Daniel Benchimol and Reinhilde Jacobs. All authors are agreed to be accountable for all aspects of the work.

## CONFLICTS OF INTEREST

The authors declare no conflicts of interest.

### 1 ETHICS STATEMENT

This material is the authors' own original work, which has not been previously published elsewhere. The paper is not currently being considered for publication elsewhere. The paper reflects the authors' own research and analysis in a truthful and complete manner. The paper properly credits the meaningful contributions of co‐authors and co‐researchers. The results are appropriately placed in the context of prior and existing research. All sources used are properly disclosed (correct citation). Literally copying of text must be indicated as such by using quotation marks and giving proper reference. All authors have been personally and actively involved in substantial work leading to the paper and will take public responsibility for its content.

## Data Availability

Data are available on request from the authors.
